# Disrupted diurnal oscillations of the gut microbiota in patients with alcohol dependence

**DOI:** 10.3389/fcimb.2023.1127011

**Published:** 2023-02-17

**Authors:** Kangqing Zhao, Zhaojun Ni, Ying Qin, Ran Zhu, Zhoulong Yu, Yundong Ma, Wenhao Chen, Qiqing Sun, Zhong Wang, Yanjing Liu, Jingwen Zhao, Wenjuan Peng, Sifan Hu, Jie Shi, Lin Lu, Hongqiang Sun

**Affiliations:** ^1^ NHC Key Laboratory of Mental Health (Peking University), Peking University Sixth Hospital, Peking University Institute of Mental Health, National Clinical Research Center for Mental Disorders (Peking University Sixth Hospital), Beijing, China; ^2^ Addiction Medicine Department, The Second People’s Hospital of Guizhou Province, Guizhou, China; ^3^ National Institute on Drug Dependence and Beijing Key Laboratory of Drug Dependence, Peking University, Beijing, China; ^4^ The State Key Laboratory of Natural and Biomimetic Drugs, Peking University, Beijing, China; ^5^ The Key Laboratory for Neuroscience of the Ministry of Education and Health, Peking University, Beijing, China

**Keywords:** alcohol dependence, diurnal oscillation, gut microbiota, dysbiosis, rhythmicity

## Abstract

**Background:**

Patients with alcohol dependence (AD) can exhibit gut dysbacteria. Dysbacteria may co-occur with disruptions of circadian rhythmicity of the gut flora, which can aggravate AD. Herein, this study aimed to investigate diurnal oscillations of the gut microbiota in AD patients.

**Methods:**

Thirty-two patients with AD, based on the Diagnostic and Statistical Manual of Mental Disorders, 4th edition, and 20 healthy subjects were enrolled in this study. Demographic and clinical data were collected by self-report questionnaires. Fecal samples at 7:00 AM, 11:00 AM, 3:00 PM, and 7:00 PM were collected from each subject. 16S rDNA sequencing was conducted. Wilcoxon and Kruskal-Wallis tests were performed to characterize alterations and oscillations of the gut microbiota.

**Results:**

We found that β-diversity of the gut microbiota in AD patients oscillated diurnally compared with healthy subjects (p = 0.01). Additionally, 0.66% of operational taxonomic units oscillated diurnally in AD patients versus 1.68% in healthy subjects. At different taxonomic levels, bacterial abundance oscillated diurnally in both groups, such as Pseudomonas and Prevotella pallens (all p < 0.05). β-diversity of the gut microbiota in AD patients with high daily alcohol consumption, high-level cravings, short AD durations, and mild withdrawal symptoms oscillated diurnally compared with other AD patients (all p < 0.05).

**Conclusion:**

The gut microbiota in AD patients exhibits disruptions of diurnal oscillation, which may provide novel insights into mechanisms of AD and the development of therapeutic strategies.

## 1 Introduction

Alcohol dependence (AD) remains a severe medical problem (De La Monte and Kril, 2014; Stockwell, 2015), but its biological mechanisms are largely unknown. There is a lack of targeted, effective treatments for AD, underscoring the vital need to understand its underlying factors. Recent evidence suggests that AD patients exhibit altered gut microbiota profiles, which, through the microbiota-gut-brain axis, is related to the severity of physiological symptoms (Mutlu et al., 2012; Leclercq et al., 2014).

Human intestinal flora comprises over 1000 species, mostly belonging to Firmicutes, Bacteroidetes, Proteobacteria, Actinobacteria, Verrucomicrobia, and Fusobacteria (Ley et al., 2008). Gut microbiota dysbiosis has been reported to be positively correlated with the misuse of alcohol (Mutlu et al., 2012), cocaine (Volpe et al., 2014), opioids (Acharya et al., 2017), methamphetamine (Xu et al., 2017), and heroin (Xu et al., 2017). Both animal and human studies have shown that AD may cause altered gut microbiota profiles (Mutlu et al., 2009; Mutlu et al., 2012; Leclercq et al., 2014; Dubinkina et al., 2017). Leclercq et al. and Dubinkina et al. reported that the abundance of Ruminococcaceae decreased, and Lachnospiraceae was enriched, with alterations of six genera and 34 species in the gut microbiota of AD patients compared with healthy subjects (Leclercq et al., 2014; Dubinkina et al., 2017). Meanwhile, another study reported that the abundance of Bacteroidetes, Verrucomicrobiae, and Clostridia was lower, and the abundance of Gammaproteobacteria was higher in AD patients (Mutlu et al., 2012). These findings show that the gut microbiota certainly plays an important role in the pathogenesis of AD.

A human study found that approximately 10% of operational taxonomic units (OTUs) of the gut microbiota exhibited circadian rhythmicity (Thaiss et al., 2014). The abundance of Firmicutes peaked during the day and reached its nadir at night. In contrast, the abundance of Bacteroidetes and Verrucomicrobia peaked at night and was lowest during the day (Thaiss et al., 2014; Zarrinpar et al., 2014). Metabolites produced by the gut microbiota fluctuate throughout the day (Thaiss et al., 2016; Tahara et al., 2018). These metabolites have various functions. For example, short-chain fatty acids and α-aminobutyric acid modulate the expression of circadian clock genes (Zwighaft et al., 2015; Tahara et al., 2018). The metabolites fluctuation helps ensure metabolic homeostasis. Disrupted rhythmicity of the gut microbiota may lead to infections, liver damage, metabolic disorders, and cancers, thereby aggravating AD (Nobs et al., 2019).

Rhythmicity-wise, the crosstalk between hosts and gut microbiota has been well-documented. The circadian rhythmicity in mammals is constituted by central clock in the suprachiasmatic nucleus of hypothalamus and peripheral clock within each cell in the body (Nobs et al., 2019). The gastro-intestinal tract features a rhythmically altering environment, which entrains the rhythmicity of gut microbiota (Nobs et al., 2019). In turn, rhythmic microbiota attachment to the gastro-intestinal epithelia could program the transcriptome oscillations of the epithelia, and additionally, metabolites produced by gut microbiota could affect the expression of hepatic clock genes (Thaiss et al., 2016). Alcohol-related disorders have been demonstrated to affect melatonin secretion through reactive oxygen species, thus leading to circadian disruption (Voigt et al., 2016), and circadian disruption can further disturb the circadian rhythmicity of the gut flora, which conversely, can also induce circadian disruption of the host, and this crosstalk may aggravate the pathogenesis of AD (Falcón and Mcclung, 2009; Thaiss et al., 2014; Zarrinpar et al., 2014; Thaiss et al., 2016; Nobs et al., 2019; Kurhaluk and Tkachenko, 2020; Meyrel et al., 2020).

Our study tested the hypothesis that diurnal oscillation of the gut microbiota is disrupted in humans with AD. Our findings may provide a theoretical basis for unraveling the mechanisms of AD and contribute to the development of new therapeutic strategies.

## 2 Material and methods

### 2.1 Subjects

Thirty-seven AD patients and 21 healthy subjects were enrolled in this study. Alcohol dependence was diagnosed based on the criteria of the *Diagnostic and Statistical Manual of Mental Disorders*, 4th edition (DSM-IV) (American Psychiatric Association, 1994), when subjects were admitted to the Guizhou 2^nd^ Provincial People’s Hospital from July 2019 to September 2019. At admission, the subjects’ medical history, demographic data, and relevant clinical data were collected.

The inclusion criteria for the AD group were the following: (1) diagnosis of AD according to the DSM-IV and (2) Han Chinese males aged 18-60 years. The exclusion criteria for the AD group were the following: (1) metabolic disorders, such as diabetes and obesity, (2) severe physical diseases, such as cardiovascular disease and infectious disease, (3) DSM-IV Axis I disorders other than alcohol and nicotine dependence, (4) night shift workers, (5) use of antibiotics, steroids, or other microbiota-modulating medications within 2 months before enrollment, and (6) abstinence longer than 1 week. The inclusion criterion for the healthy control (HC) group was Han male aged 18-60 years. The exclusion criteria for the HC group were identical to the AD group except DSM-IV Axis I disorders other than alcohol and nicotine dependence, where AD was excluded.

All enrolled subjects were in the hospital the entire time before the completion of four fecal sample collections and had the same meals provided by the hospital. The details of medications that were prescribed during fecal sampling are provided in **Supplementary Table S1**.

### 2.2 Assessment tools for AD severity and nicotine dependence

The Pennsylvania Alcohol Craving Scale (PACS) (Flannery et al., 1999), Revised Clinic Institute Alcohol Withdrawal Syndrome Assessment Scale (CIWA-Ar) (Sullivan et al., 1989), Fagerstrom Test of Nicotine Dependence (Heatherton et al., 1991), and self-reported alcohol-related questionnaires were used to measure the level of alcohol craving, severity of withdrawal, AD duration, and daily alcohol consumption.

### 2.3 Subgrouping of AD patients

Based on the disease duration, daily consumption, withdrawal severity according to CIWA-Ar, and craving levels according to PACS, AD patients were subdivided into a series of subgroups. With regard to daily alcohol consumption, AD patients were divided into the high standard drinks (HSD) group (≥ 15 standard drinks; 16 subjects) and the low standard drinks (LSD) group (< 15 standard drinks; 16 subjects) (Whelan, 2002). With regard to the duration of AD, AD patients were divided into the long courses (LC) group (AD duration ≥ 10 years; 18 subjects) and the short courses (SC) group (AD duration < 10 years; 14 subjects) (Schuckit and Smith, 2011). With regard to the severity of withdrawal symptoms, AD patients were divided into the high CIWA-Ar (HCW) group (CIWA-Ar ≥ 16; 18 subjects) and the low CIWA-Ar (LCW) group (CIWA-Ar < 16; 14 subjects) (Zhuo et al., 2009). With regard to craving levels, based on the distribution of PACS scores in our study, AD patients were divided into the high PACS (HPA) group (PACS ≥ 6; 16 subjects) and the low PACS (LPA) group (PACS < 6; 16 subjects).

### 2.4 Gut microbiota analysis

Four fecal samples were collected from each subject at 7:00 AM, 11:00 AM, 3:00 PM, and 7:00 PM within 4 days of enrollment. The four fecal samples collected from 32 AD patients and 20 HC subjects were used for the analysis, the fecal samples at a different time point rather than 7:00 AM, 11:00 AM, 3:00 PM, and 7:00 PM were not collected for the analysis and the details are presented in **Supplementary Figure S1** and **Table S2**.

According to the 12th edition of the Manual of Procedures for the Human Microbiome Project (Mcinnes and Cutting, 2010), fecal samples were collected in a sterile tube, frozen in a liquid nitrogen flash-freezer, and stored at -80°C within 30 min of collection. For 16S rDNA sequencing, polymerase chain reaction (PCR) amplification was performed, spanning the V4 region of the 16S rRNA gene. The PCR products were sequenced using 2 × 250 bp paired-end sequencing (Illumina HiSeq). Further details of the PCR methods are provided in the Supplementary Information.

#### 2.4.1 DNA extraction

The microbial community DNA was extracted using a MagPure Stool DNA KF Kit B (Magen, China) following the manufacturer’s instructions. DNA was quantified with a Qubit Fluorometer by using a Qubit^®^ dsDNA BR Assay Kit (Invitrogen, USA), and the quality was checked by electrophoresing aliquots on a 1% agarose gel.

#### 2.4.2 Library construction

The variable region V4 of the bacterial 16S rRNA gene was amplified with the degenerate PCR primers 515F (5’-GTGCCAGCMGCCGCGGTAA-3’) and 806R (5’-GGACTACHVGGGTWTCTAAT-3’). Both the forward and reverse primers were tagged with Illumina adaptor, pad and linker sequences. PCR enrichment was performed in a 50 μL reaction containing 30 ng of template, fusion PCR primer and PCR master mix. PCR cycling conditions were as follows: 95°C for 3 minutes; 30 cycles of 95°C for 45 seconds, 56°C for 45 seconds, and 72°C for 45 seconds; and final extension for 10 minutes at 72°C. The PCR products were purified using Agencourt AMPure XP beads and eluted in elution buffer. Libraries were qualified by an Agilent Technologies 2100 bioanalyzer. The validated libraries were used for sequencing on the Illumina HiSeq 2500 platform (BGI, Shenzhen, China) following the standard pipelines of Illumina, which generated 2 × 250 bp paired-end reads.

#### 2.4.3 Sequencing and bioinformatics analysis

##### 2.4.3.1 Data filtering

Raw data are filtered to generate high quality clean reads as follows (Magoc and Salzberg, 2011): 1) Truncate reads whose average phred quality values are lower than 20 over a 30 bp sliding window will be truncated. Remove reads whose length are 75% of their original lengths after truncation; 2) Remove reads that are contaminated by adapter sequences; 3) Remove reads with ambiguous base (N base); 4) Remove low-complexity reads. iTools Fqtools fqcheck (v.0.25) is used for quality control, cutadapt (v.2.6) is used to remove adaptor, and readfq (v1.0) is used for flitering.

##### 2.4.3.2 Tags connection

If paired-end reads overlap with each other, then a consensus sequence will be generated by FLASH (Fast Length Adjustment of Short reads, v1.2.11) (He et al., 2013). Details are given as follows: 1) Minimum overlapping length:15 bp; 2) Mismatching ratio of overlapped region: 0.1.

Meanwhile, the data are rarefied to ensure that there is no deviation in the calculation of species abundance because of the differences between samples.

##### 2.4.3.3 OTU clustering

Tags are clustered to OTU with USEARCH (v7.0.1090), details are given as follows: 1) Tags are clustered into OTU with a 97% threshold by UPARSE, where the unique OTU representative sequences can be obtained; 2) Chimeras are filtered by UCHIME (v4.2.40), and chimeras in OTU are screened and filtered by mapping to gold database(v20110519); 3) All tags are mapped to OTU representative sequences using USEARCH GLOBAL to calculate OTU abundance table.

##### 2.4.3.4 OTU taxonomy annotation

OTU representative sequences are aligned against the Greengene V201305 database for taxonomic annotation by RDP classifer (v2.2) software (sequence identity is set to be 0.6). All the OTUs will be annotated accordingly and the OTUs that are not annotated will be removed.

##### 2.4.3.5 Alpha diversity analysis

Alpha diversity refers to the analysis of species diversity in a sample and is measured by chao index, ace index, shannon index, simpson index, etc. It’s estimated by estimated by MOTHUR (version 1.31.2) (Schloss et al., 2009).

##### 2.4.3.6 Beta diversity analysis

Beta diversity analysis (Lozupone and Knight, 2005; Lozupone et al., 2007; Lozupone et al., 2011) was used to evaluate differences of samples in species complexity. Beta diversity analysis was done by software QIIME(v1.80).

##### 2.4.3.7 Function prediction

PICRUSt2 (Phylogenetic Investigation of Communities by Reconstruction of Unobserved States, v2.3.0-b) (Douglas et al., 2019) predicts the KEGG functions abundance of microbial community based on marker gene sequencing profiles.

### 2.5 Statistical analysis

The statistical analyses of sociodemographic and clinical data were performed using SPSS 23.0 software (IBM Corp). Education years and BMI from all the included subjects met normality of the data and equal variance, determined by the Kolmogorov–Smirnov and Levene tests, respectively, and independent *t*-tests were used to compare the AD and HC groups; FTND scores and ages from all the subjects did not meet normality of the data and equal variance, and the Kruskal-Wallis test was performed. For marital status and occupation, the *χ^2^* test was performed. To test differences in the gut microbiota between the AD and HC groups, the Wilcoxon test was performed for the α-diversity, β-diversity, relative abundances of bacteria and KEGG pathways. To determine diurnal oscillations, because of the limited number of time points, Jonckheere-Terpstra-Kendall analysis and cosine analysis were not feasible. Therefore, to determine the diurnal oscillation, based on the fecal samples at the four different time points, Kruskal-Wallis test and Dunn’s multiple-comparison test by Prism 8.00 software (GraphPad, La Jolla, CA, USA) and R 3.1.1. software were used for the OTUs, β-diversity, relative abundances of bacteria at different taxonomic levels and KEGG pathways, and Principal coordinate analysis (PCoA) was performed by QIIME (version 1.8.0) (Caporaso et al., 2010) based on weighted UniFrac distance. Microbiota-related data are expressed as the mean ± SEM.

### 2.6 Ethics statement

The study protocol was approved by the Ethics Committee of Peking University Sixth Hospital on April 17, 2019. All enrolled subjects signed an informed consent form.

## 3 Results

### 3.1 Sociodemographic and clinical characteristics of the subjects

We statistically analyzed clinical baselines between the AD and HC groups. On the aspect of age, occupation, education years, marital status, BMI and FTND scores, no significant differences were found between AD patients and healthy subjects. However, the sleep quality of the AD group was statistically worse than the HC group (**Table 1**). The duration of AD, daily consumption of alcohol, craving levels and withdrawal severity were also elaborated in **Table 1**.

**Table 1:** Demographic and clinical data for the AD and HC groups.

Variable		AD	HC	*χ* ^2^/*t*	*p*
Number		32	20	/	*/*
Age (years)		44 (35.75, 51.50)	45 (26.25, 51.00)	0.00	0.95
Occupation	Employed	23 (71.88%)	14 (70%)	0.02	1.00
	Unemployed	9 (28.12%)	6 (30%)		
Education (years)		7.31 ± 4.31	8.25 ± 4.23	-0.77	0.45
Marital status	Married	27 (84.38%)	14 (70%)	1.53	0.30
	Single, Divorced, or Widowed	5 (15.62%)	6 (30%)		
BMI (kg/m^2^)		21.75 ± 2.17	22.69 ± 3.03	-1.03	0.20
FTND		4.00 (2.00, 6.00)	4.00 (0.00, 4.00)	0.00	0.98
PSQI		7.00 (4.00, 15.75)	2.00 (1.00, 4.00)	8.70	**
Disease duration (years)		10.00 (5.00, 17.00)	/	/	/
Daily consumption (standard drinks)		15 (7.00, 23.00)	/	/	/
PACS		5.50 (3.00, 9.00)	/	/	/
CIWA-Ar		17.16 ± 8.00	/	/	/

Data with a normal distribution and equal variance are expressed as mean ± SD. Data without normality and equal variance are expressed as medians and interquartile ranges. AD, alcohol dependence; HC, healthy control; BMI, Body Mass Index; FTND, Fagerstrom Test of Nicotine Dependence; PSQI, Pittsburg Sleep Quality Index; PACS, Pennsylvania Alcohol Craving Scale; CIWA-Ar, Revised Clinic Institute Alcohol Withdrawal Syndrome Assessment Scale. **p < 0.01.

### 3.2 Alterations of the gut microbiota in AD patients

**Figure 1:**
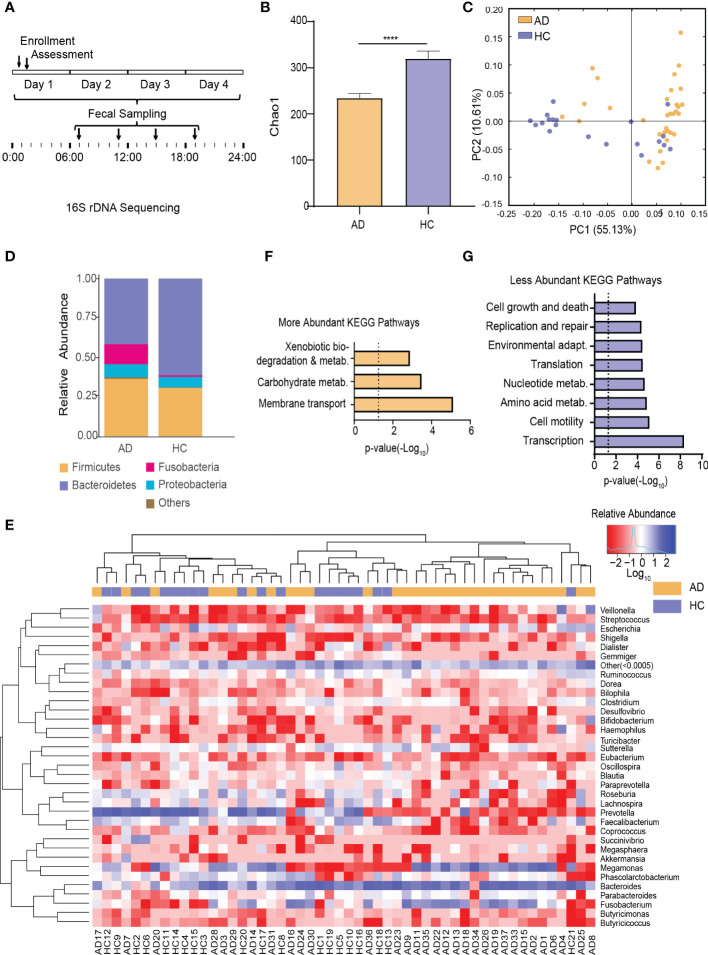
Alterations of the Gut Microbiota in AD Patients. **(A)** Schematic diagram of sampling times of the gut microbiota following enrollment. **(B)** α-Diversity of the gut microbiota in the AD and HC groups. **(C)** β-Diversity of the gut microbiota in the AD and HC groups. **(D)** Stacked bar graph of the average relative abundance of each phylum in the AD and HC groups. **(E)** Heatmap of the most prevalent genera in the gut microbiota in the AD and HC groups. Columns correspond to the subjects. Groups are denoted with a color bar on top. The heatmap shows taxa with a relative abundance ≥ 0.05%. Hierarchical clustering was performed using the Euclidean metric and complete linkage. **(F)** KEGG pathways with higher relative abundance in the AD group than in the HC group. **(G)** KEGG pathways with lower relative abundance in the AD group than in the HC group. ****p < 0.0001. AD, alcohol dependence; HC, healthy control; KEGG, Kyoto Encyclopedia of Genes and Genomes.

This trial collected 52 × 4 fecal samples at the four different time points following enrollment (**Figure 1A**). The number of clean reads per sample is 71090*2~75123*2. To test for alterations of the gut microbiota in AD patients reported previously in human studies (Mutlu et al., 2012; Leclercq et al., 2014; Dubinkina et al., 2017), fecal samples taken at 7:00 AM (i.e., when most subjects normally defecated spontaneously) were analyzed. α-Diversity of the gut microbiota was significantly different between the AD and HC groups (*p* < 0.0001, Wilcoxon test; **Figure 1B**). Based on weighted UniFrac distance, the gut microbiota in the AD group differed from the HC group (*p* < 0.001, Wilcoxon test; **Figure 1C**). Further analyses showed that at the bacterial phylum level, the AD group exhibited an increase in the relative abundance of Fusobacteria and Tenericutes (all *p* < 0.05, Wilcoxon test) and a decrease in the relative abundance of Bacteroidetes, Lentisphaerae, Synergistetes, and unclassified bacteria compared with the HC group (all *p* < 0.05, Wilcoxon test; **Figure 1D**, **Supplementary Figure S2A-C**). At the class level, Bacteroidia, Lentisphaeria, and Synergistia were less abundant (all *p* < 0.05, Wilcoxon test), whereas Fusobacteriia, Actinobacteria, and Mollicutes were more abundant in the AD group (all *p* < 0.05, Wilcoxon test; **Supplementary Figure S2D**). At the order level, Fusobacteriales, Rickettsiales, and Actinomycetales were enriched (all *p* < 0.05, Wilcoxon test), whereas Bacteroidales, Synergistales, and Victivallales were depleted in the AD group (all *p* < 0.05, Wilcoxon test; **Supplementary Figure S2E**). At the family level, the AD group had higher levels of Fusobacteriaceae, Veillonellaceae, Actinomycetaceae, Eubacteriaceae, Porphyromonadaceae, and Pseudomonadaceae (all *p* < 0.05, Wilcoxon test) and lower levels of Ruminococcaceae, Lachnospiraceae, Paraprevotellaceae, Peptococcaceae, Barnesiellaceae, Mogibacteriaceae, Christensenellaceae, Dehalobacteriaceae, Bacteroidaceae, Clostridiaceae, Dethiosulfovibrionaceae, and Victivallaceae (all *p* < 0.05, Wilcoxon test; **Supplementary Figure S2F**). The AD group exhibited increases in the relative abundance of 15 genera (all *p* < 0.05, Wilcoxon test; **Figure 1E**, **Supplementary Table S3**) and 16 species (all *p* < 0.05, Wilcoxon test; **Supplementary Figure S2G**, **Table S4**). In contrast, the AD group exhibited decreases in the relative abundance of 15 genera (all *p* < 0.05, Wilcoxon test; **Figure 1E**, **Supplementary Table S3**) and 14 species (all *p* < 0.05, Wilcoxon test; **Supplementary Figure S2G**, **Table S4**).

Among the detected KEGG pathways in the AD group, the relative abundance of KEGG pathways involved in xenobiotics and carbohydrate metabolism was higher (**Figure 1F**), whereas the relative abundance of KEGG pathways involved in replication and repair, transcription, translation, nucleotide metabolism, cell motility, cell growth and death, environmental adaptation, and amino acid metabolism were lower (**Figure 1G**) compared with the HC group (**Supplementary Figure S2H**).

### 3.3 Diurnal oscillation of the gut microbiota in the AD and HC groups

**Figure 2:**
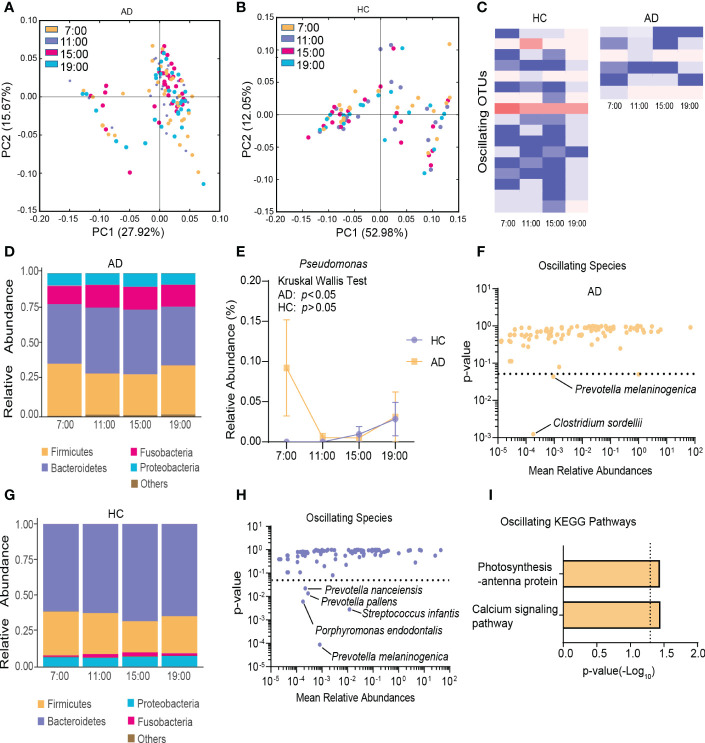
Diurnal oscillation of the gut microbiota. **(A)** Principal coordinate analysis of weighted UniFrac distance in the AD group and Kruskal-Wallis test showing statistically significant oscillation across the four time points in the AD group (*p* < 0.05). **(B)** Principal coordinate analysis of weighted UniFrac distance in the HC group and Kruskal-Wallis test showing no statistically significant oscillation across the four time points in the HC group. **(C)** Heatmap of OTUs that oscillated with *p* < 0.05 in the AD and HC groups. **(D)** Stacked bar graph of the average relative abundance of each phylum in the AD group. **(E)** Relative abundance of *Pseudomonas* at four different time points in each group. **(F)** Species that showed diurnal oscillation in the AD group. **(G)** Stacked bar graph of the average relative abundance of each phylum in the HC group. **(H)** Species that showed diurnal oscillation in the HC group. **(I)** KEGG pathways that oscillated in the AD group. The data are expressed as the mean ± SEM. AD, alcohol dependence; HC, healthy control; KEGG, Kyoto Encyclopedia of Genes and Genomes; OTUs, operational taxonomic units.

To characterize diurnal oscillation of the gut microbiota, taxonomic analysis of the fecal microbiota at the four different time points was performed. In the AD group, based on weighted UniFrac distance, β-diversity among the four time points exhibited robust oscillation (*p* = 0.01, Kruskal-Wallis test; **Figure 2A**). In the HC group, the oscillation of β-diversity was completely absent (**Figure 2B**). Approximately 1.68% of OTUs in the HC group and 0.66% of OTUs in the AD group exhibited robust oscillation (**Figure 2C**). Additionally, the abundance of bacteria showed different oscillation patterns between the AD and HC groups.

In the AD group at the phylum level, Cyanobacteria exhibited diurnal fluctuations among the four different time points (*p* < 0.05, Kruskal-Wallis test; **Figure 2D**, **Supplementary Figure S3A**). At the class and order levels, none exhibited diurnal fluctuations. At the family, genus, and species levels, Pseudomonadaceae, *Pseudomonas, Clostridium sordellii*, and *Prevotella melaninogenica* exhibited diurnal fluctuations (all *p* < 0.05, Kruskal-Wallis test; **Figure 2E, F**, **Supplementary Figure S3B**).

In the HC group at the phylum level, no significant diurnal fluctuations were found among the four different time points (**Figure 2G**). However, Firmicutes showed a trend toward a significant fluctuation (*p* = 0.06, Kruskal-Wallis test). At the class level, Flavobacteriia exhibited diurnal fluctuations (*p* < 0.01, Kruskal-Wallis test; **Supplementary Figure S3C**). At the order level, Rickettsiales, Flavobacteriales, and Neisseriales exhibited diurnal fluctuations (all *p* < 0.05, Kruskal-Wallis test; **Supplementary Figure S3D**). At the family, genus, and species levels, Flavobacteriaceae, Neisseriaceae, Streptococcaceae, *Porphyromonas*, *Capnocytophaga*, *Moryella*, *Tannerella*, *Atopobium*, *Prevotella melaninogenica*, *Streptococcus infantis*, *Porphyromonas endodontalis*, *Prevotella pallens*, and *Prevotella nanceiensis* exhibited diurnal fluctuations (all *p* < 0.05, Kruskal-Wallis test; **Figure 2H**, **Supplementary Figure S3E, F**).

Among the detected KEGG pathways in the AD group, the relative abundance of “calcium signaling pathway” and “photosynthesis-antenna protein” oscillated among the four time points (**Figure 2I**, **Supplementary Figure S3G**), but none oscillated in the HC group (**Supplementary Figure S3H**).

### 3.4 Different oscillation patterns of the gut microbiota in AD patients with different daily alcohol consumption

**Figure 3:**
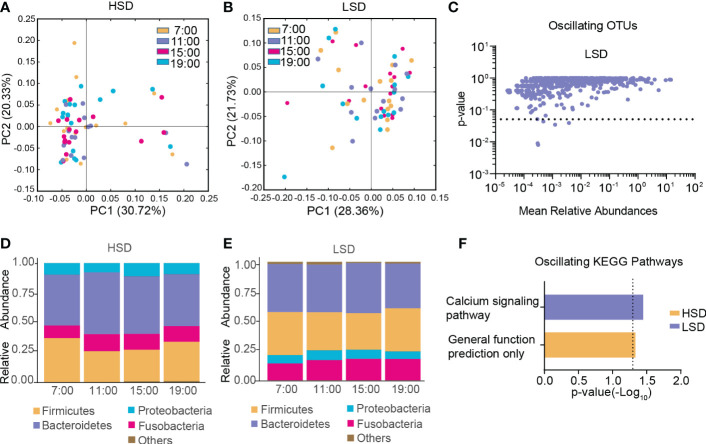
Diurnal oscillation of the gut microbiota in AD patients with different daily alcohol consumption. **(A)** Principal coordinate analysis of weighted UniFrac distance in the HSD group. **(B)** Principal coordinate analysis of weighted UniFrac distance in the LSD group. **(C)** Operational taxonomic units that showed diurnal oscillation in the LSD group. **(D)** Stacked bar graph of the average relative abundance of each phylum in the HSD group. **(E)** Stacked bar graph of the average relative abundance of each phylum in the LSD group. **(F)** KEGG pathways that oscillated in the HSD and LSD groups. HSD, high-standard-drink (≥ 15 standard drinks); KEGG, Kyoto Encyclopedia of Genes and Genomes; LSD, low-standard-drink (< 15 standard drinks); OTUs, operational taxonomic units.

In the HSD group, β-diversity of the gut microbiota among the four different time points exhibited robust oscillation (*p < 0.05*, Kruskal-Wallis test; **Figure 3A**), and no OTUs exhibited diurnal fluctuation. In the LSD group, no oscillation of β-diversity was observed (**Figure 3B**), and approximately 0.63% of OTUs displayed diurnal fluctuation (**Figure 3C**). At all taxonomic levels, no bacteria oscillated diurnally in either group (**Figure 3D, E**). Furthermore, among the detected KEGG pathways, the relative abundance of “general function prediction only” exhibited diurnal oscillation in the HSD group, and the relative abundance of “calcium signaling pathway” exhibited diurnal oscillation in the LSD group (**Figure 3F**).

### 3.5 Different oscillation patterns of the gut microbiota in AD patients with different disease durations

**Figure 4:**
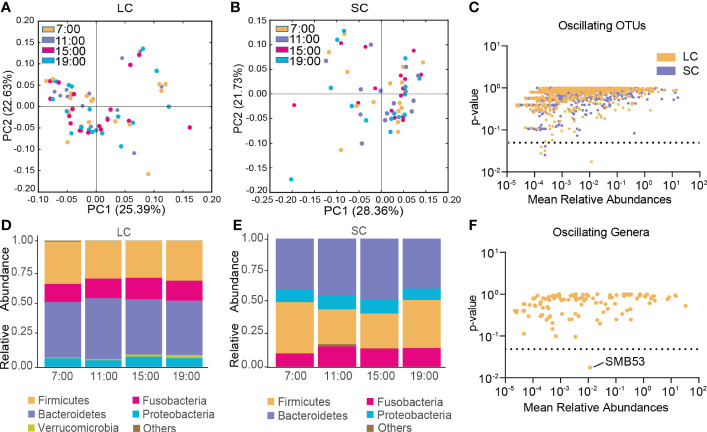
Diurnal oscillation of the gut microbiota in AD patients with different disease durations. **(A)** Principal coordinate analysis of weighted UniFrac distance in the LC group and Kruskal-Wallis test showing no statistically significant oscillation across the four time points in the LC group. **(B)** Principal coordinate analysis of weighted UniFrac distance in the SC group and Kruskal-Wallis test showing statistically significant oscillation across the four time points in the SC group (*p* < 0.05). **(C)** Operational taxonomic units that showed diurnal oscillation in each group. **(D)** Stacked bar graph of the average relative abundance of each phylum in the LC group. **(E)** Stacked bar graph of the average relative abundance of each phylum in the SC group. **(F)** Genera that showed diurnal oscillation in the LC group. LC, long course (≥ 10 years of disease duration; OTUs, operational taxonomic units; SC, short course (< 10 years of disease duration).

In the LC group, β-diversity of the gut microbiota exhibited no diurnal oscillation (**Figure 4A**), and β-diversity displayed diurnal oscillation in the SC group (p < 0.05, Kruskal-Wallis test; **Figure 4B**). Approximately 0.50% of OTUs displayed diurnal oscillation in the LC group and approximately 0.26% of OTUs exhibited diurnal oscillation in the SC group (**Figure 4C**). In both groups at the phylum, class, order, family, and species levels, the gut microbiota exhibited no statistically significant diurnal oscillation (**Figure 4D, E**). At the genus level, SMB53 exhibited diurnal oscillation in the LC group (**Figure 4F**). Among the detected KEGG pathways, none displayed diurnal oscillation in either group.

### 3.6 Different oscillation patterns of the gut microbiota in AD patients with different withdrawal severities

**Figure 5:**
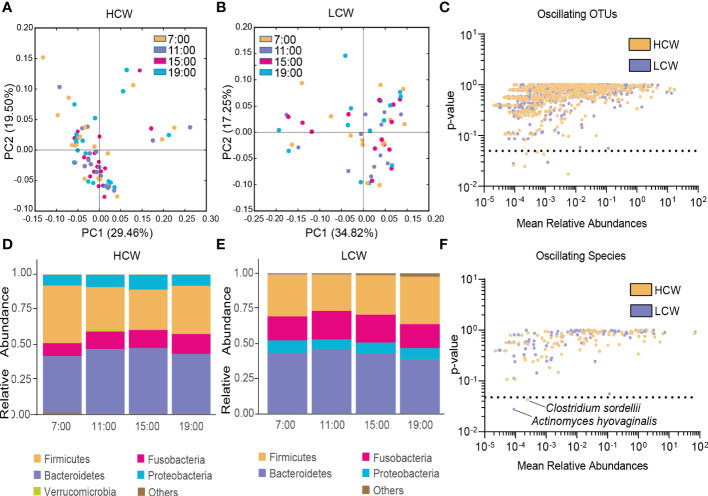
Diurnal oscillation of the gut microbiota in AD patients with different severities of withdrawal. **(A)** Principal coordinate analysis of weighted UniFrac distance in the HCW group and Kruskal-Wallis test showing statistically significant oscillation across the four time points in the HCW group (*p* < 0.001). **(B)** Principal coordinate analysis of weighted UniFrac distance in the LCW group and Kruskal-Wallis test showing statistically significant oscillation in the LCW group (*p* < 0.001). **(C)** Operational taxonomic units that showed diurnal oscillation in each group. **(D)** Stacked bar graph of the average relative abundance of each phylum in the HCW group. **(E)** Stacked bar graph of the average relative abundance of each phylum in the LCW group. **(F)** Species that showed diurnal oscillation in each group. HCW, high CIWA-Ar (CIWA-Ar score ≥ 16); LCW, low CIWA-Ar (CIWA-Ar score < 16); OTUs, operational taxonomic units.

In both the HCW and LCW groups, β-diversity of the gut microbiota displayed diurnal oscillation (both *p* < 0.001, Kruskal-Wallis test; **Figure 5A, B**). Approximately 0.75% of OTUs in the HCW group, and 0.39% of OTUs in the LCW group displayed diurnal oscillation (**Figure 5C**). At the phylum, class, order, family, and genus levels, the gut microbiota did not exhibit diurnal oscillation in either group (**Figure 5D, E**). At the species level, *Clostridium sordellii* exhibited diurnal oscillation in the HCW group, and *Actinomyces hyovaginalis* exhibited diurnal oscillation in the LCW group (**Figure 5F**). Among the detected KEGG pathways, none displayed diurnal oscillation in either group.

### 3.7 Different oscillation patterns of the gut microbiota in AD patients with different craving levels

**Figure 6:**
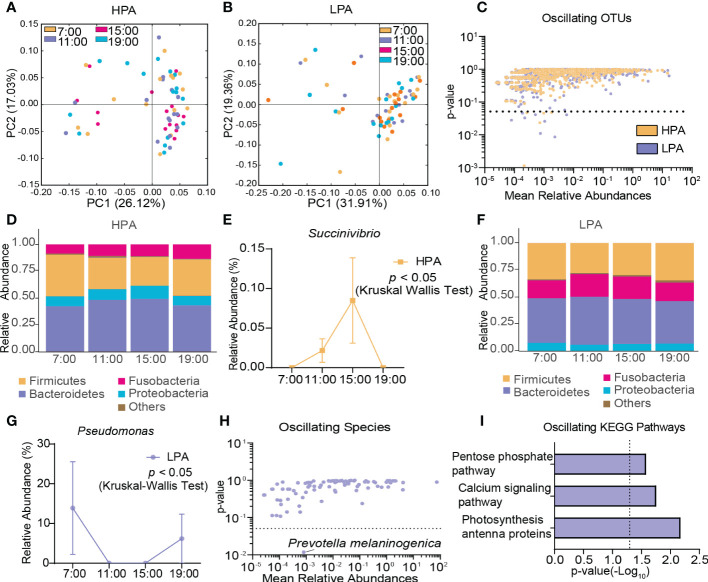
Diurnal oscillation of the gut microbiota in AD patients with different craving levels. **(A)** Principal coordinate analysis of weighted UniFrac distance in the HPA group and Kruskal-Wallis test showing statistically significant oscillation across the four time points in the HPA group (*p* < 0.05). **(B)** Principal coordinate analysis of weighted UniFrac distance in the LPA group and Kruskal-Wallis test showing no statistically significant oscillation across the four time points in the LPA group. **(C)** Operational taxonomic units that showed diurnal oscillation in each group. **(D)** Stacked bar graph of the average relative abundance of each phylum in the HPA group. **(E)** Diurnal oscillation of the relative abundance of *Succinivibrio.* **(F)** Stacked bar graph of the average relative abundance of each phylum in the LPA group. **(G)** Diurnal oscillation of the relative abundance of *Pseudomonas*. **(H)** Species that showed diurnal oscillation in each group. **(I)** KEGG pathways that oscillated in LPA patients. HPA, high-PACS (PACS score ≥ 6); KEGG, Kyoto Encyclopedia of Genes and Genomes; LPA, low-PACS (PACS score < 6); OTUs, operational taxonomic units. The data are expressed as the mean ± SEM.

In the HPA group, β-diversity of the gut microbiota displayed diurnal oscillation (*p* < 0.05, Kruskal-Wallis test; **Figure 6A**), whereas the fluctuation of β-diversity was lost in the LPA group (**Figure 6B**). Approximately 0.62% of OTUs in the HPA group and 1.04% of OTUs in the LPA group exhibited diurnal oscillation (**Figure 6C**). In the HPA group at the phylum level, TM7 exhibited diurnal oscillation (**Figure 6D**, **Supplementary Figure S4A**). At the class level, TM7_3 exhibited diurnal oscillation (**Supplementary Figure S4B**). No order, family, or species showed diurnal oscillation. At the genus level, three genera exhibited diurnal oscillation (*Sarcina*, *Anaerococcus*, and *Succinivibrio*; **Figure 6E**, **Supplementary Figure S4C, D**). In the LPA group at the phylum level, Cyanobacteria exhibited diurnal oscillation (**Figure 6F**, **Supplementary Figure S4E**). At the class level, none exhibited diurnal oscillation. At the order level, the relative abundance of Pseudomonadales and Rickettsiales exhibited diurnal oscillation (**Supplementary Figure S4F, G**). At the family, genus, and species levels, Pseudomonadaceae, *Pseudomonas*, and *Prevotella melaninogenica* exhibited diurnal oscillation (**Figure 6G, H**, **Supplementary Figure S4H, I**). Among the detected KEGG pathways, the relative abundance of “photosynthesis-antenna protein,” “calcium signaling pathway,” and “pentose phosphate pathway” exhibited diurnal oscillation in the LPA group (**Figure 6I**), whereas no KEGG pathways exhibited diurnal oscillation in the HPA group.

## 4 Discussion

Previous studies reported that AD patients may have altered gut microbiota profiles including lower levels of *Bacteroidetes*, *Prevotella*, *Faecalibacterium*, and *Coprococcus* (Mutlu et al., 2012; Leclercq et al., 2014; Dubinkina et al., 2017), which is consistent with our observations. We also found an increase in the abundance of Fusobacteria, which may play a protective role in liver function (Puri et al., 2018). Our study also showed that bacteria with anti-inflammatory properties and nutritious functions were less abundant in AD patients than in healthy subjects. For example, AD patients had lower levels of bacteria from Ruminococcaceae, especially *Faecalibacterium prausnitzii* with anti-inflammatory properties (Leclercq et al., 2014), and *Prevotella* providing folate and thiamine in humans (Yatsunenko et al., 2012). In contrast, such pathogens as *Pseudomonas* and *Actinomyces* were more abundant in AD patients (Könönen and Wade, 2015; Azam and Khan, 2019). Additionally, richness of gut flora was lower in AD patients than in healthy subjects and negatively correlated with the level of inflammation (Le Chatelier et al., 2013). These findings indicate that the gut microbiota tilts toward dysbiosis and exacerbates inflammation in AD. Meanwhile, we found that KEGG pathways related to energy metabolism, growth, and environmental adaptation were less abundant in AD patients, whereas KEGG pathways involved in detoxification (including xenobiotic metabolism) were more abundant. Therefore, the gut microbiota in AD patients may face a lack of nutrients, toxic effects, etc.

Previous studies have shown that up to 10% of OTUs and 20% of species exhibit cyclical fluctuations in humans (Thaiss et al., 2014), the gut microbiota exhibited less fluctuation during the daytime (Thaiss et al., 2014), and the abundance of many bacteria showed their greatest differences between day and night (Thaiss et al., 2014; Zarrinpar et al., 2014). In our study, among the four different time points, β-diversity of the gut microbiota in healthy subjects showed no significant diurnal oscillation, and 1.68% of OTUs and 5.77% of species exhibited diurnal oscillation in healthy subjects. These findings indicate that the extent of diurnal oscillation of the gut microbiota is less than that of 24-h oscillation.

Our study showed that compared with healthy subjects, more pathogenic strains (e.g., Cyanobacteria and Pseudomonadaceae) exhibited diurnal oscillation, and less beneficial strains (e.g., *Prevotella pallens*) exhibited diurnal oscillation in AD patients. Most of these bacteria overlapped with the ones causing alterations of the gut microbiota in AD patients. Meanwhile, β-diversity in AD patients exhibited robust diurnal oscillation, and approximately 0.66% of OTUs and 2.44% of species exhibited diurnal oscillation. The different oscillating pattern of gut microbiota in AD patients, compared with healthy subjects, reflected the altered circadian rhythmicity of the gut microbiota, which may predispose the gut microbiota to incapability of tackling the varying environment. The gut microbiota has been demonstrated to affect the transcriptome through epigenetic processes. Vitamin B produced by gut microbiota could affect the methylation of DNA, and acetyl-CoA and NAD^+^ involved in epigenetic processes are also secreted by gut microbiota (Paul et al., 2015; Nobs et al., 2019). Additionally, gut microbiota has also been shown to regulate the bi- and tri-methylation of histone H3 at lysine 4 and the acetylation of histone H3 at lysine 27, which profoundly change the epigenetic landscape (Thaiss et al., 2016). Many aforementioned pathogenic bacteria enriched in AD patients could disturb the expression of clock genes (Wang et al., 2017).

Among the detected KEGG pathways in AD patients, only “calcium signaling pathway” and “photosynthesis-antenna protein” exhibited diurnal oscillation. The oscillation of “photosynthesis-antenna protein” may reflect the oscillation of cyanobacteria because cyanobacteria are photosynthetic. Oscillation of the “calcium signaling pathway” remains unexplained, but genome-wide association studies have demonstrated that gene polymorphisms of the calcium signaling pathway are related to AD susceptibility (Li et al., 2014).

Compared with AD patients with low daily alcohol consumption, the gut microbiota in AD patients with high daily alcohol consumption exhibited diurnal oscillation, which may indicate the greater disruption of rhythmicity of the gut microbiota. Daily alcohol consumption is negatively correlated with blood levels of GLP-1 (Traversy and Chaput, 2015), and its decrease further reduces the secretion of insulin. Furhtermore, insulin can affect the expression of clock genes, such as *Per2* (Tahara et al., 2011). Therefore, the increasing alcohol consumption may lead to dysregulation of the expression of clock genes in intestinal epithelia the gut microbiota colonizes. Thus, the gut microbiota may exhibit altered oscillation patterns. Additionally, the calcium signaling pathway exhibited diurnal oscillation in AD patients who consumed less alcohol, probably because genes in the calcium signaling pathway could reduce alcohol consumption (Li et al., 2014).

The gut microbiota in AD patients with short duration exhibited robust oscillation compared with AD patients with long duration. Long-term alcohol consumption may favor the growth of bacteria capable of metabolizing alcohol (Dubinkina et al., 2017). Therefore, the gut microbiota in AD patients with long duration is more stable. Moreover, the duration of AD has been positively correlated with blood levels of interleukin-5 (IL-5) and IL-10 (Yen et al., 2017). BMAL1 can downregulate the expression of IL-5, the elevation of which induces the disruption of *Bmal1* expression (Zaslona et al., 2017). IL-10 has anti-inflammatory properties and is mostly produced by type 3 innate lymphoid cells, which could affect rhythmicity of the gut microbiota. These may help explain why different durations of AD affect oscillation patterns of the gut microbiota.

In AD patients, mild withdrawal symptoms are associated with more pronounced gut microbiota oscillation. Imbalance of the hypothalamic-pituitary-adrenal axis could lead to withdrawal symptoms (Becker, 2012). Higher blood cortisol levels are associated with severer withdrawal symptoms or vice versa (Stephens and Wand, 2012). Glucocorticoid-response elements are involved in regulation domains of clock genes. Lower blood levels of cortisol could inhibit *REV-ERBα*, affecting the delayed expression of *CRY1* (Reddy et al., 2007), which may disrupt the molecular clock (Partch et al., 2014). These findings may help explain why withdrawal severity affected gut microbiota oscillation.

More pronounced oscillation of the gut microbiota was related to higher levels of craving. Blood levels of IL-1β were higher in AD patients with strong craving (Yen et al., 2017). IL-1β enhances the expression of *Per2* (Dudek et al., 2017), which may alter oscillation patterns of the gut microbiota. Additionally, the “calcium signaling pathway,” “photosynthesis-antenna protein,” and “pentose phosphate pathway” showed diurnal fluctuations only in AD patients with low craving levels. Calcium signaling is involved in regulating various brain functions (Li et al., 2014), and thus may lower craving. The reason for oscillation of the “photosynthesis-antenna protein” pathway remains obscure. The pentose phosphate pathway is a main pathway that generates NADPH, whose oscillation may reflect more antioxidants (Christodoulou et al., 2018).

## 5 Limitations

This study did not cover the nocturnal time-points for samples collection, and only diurnal oscillation of gut microbiota is demonstrated to reflect its rhythmicity. Meanwhile, during the time of fecal samples collection, for a short time, some AD patients took a small dosage of olanzepine, quetiapine, risperidone, fluvoxamine and valproate. The studies concerning the effects these medications have on the gut microbiota are inconsistent (Pełka-Wysiecka et al., 2019; Skonieczna-Żydecka et al., 2019), and have not been thoroughly investigated. Given that these medications were prescribed only once or twice at a small dosage, we did not exclude these subjects. Furthermore, little biological markers were monitored and no intervention was adopted in this study. Future studies should focus more on modulating the rhythmicity of gut microbiota.

## 6 Conclusion

Our study has shown that the gut microbiota, besides alterations of its makeup, in AD patients exhibits disruptions of rhythmicity, exemplified by enhanced diurnal oscillation of β-diversity and lower percentage of diurnally oscillating OTUs. Furthermore, AD patients with high daily consumptions, high craving levels, short courses and mild withdrawal symptoms exhibit more pronounced disruption of diurnal oscillation of the gut microbiota, and AD patients with these features may show greater propensity for diseases related to disruption of the gut microbiota rhythmicity.

## Data availability statement

The datasets presented in this study can be found in online repositories. The names of the repository/repositories and accession number(s) can be found below: SRA database, PRJNA905862.

## Ethics statement

The studies involving human participants were reviewed and approved by the Ethics Committee of Peking University Sixth Hospital. The patients/participants provided their written informed consent to participate in this study.

## Author contributions

HS has full access to all data and takes responsibility for integrity of the data and accuracy of the analysis. Study concept and design: KZ, HS, ZN, YM, RZ, ZY, WC and SF. Performing the experiments: KZ, YQ, YL, JZ, and WP. Acquisition, analysis, or interpretation of data: KZ, ZN, RZ, QS, ZW, and ZY. Critical revision of the manuscript for important intellectual content: All authors. Statistical analysis: KZ and RZ. Obtained funding: HS. Study supervision: HS, JS and LL. All authors contributed to the article and approved the submitted version.
