# Supplementary material for: Disrupted diurnal oscillations of the gut microbiota in patients with alcohol dependence
Source: Front Cell Infect Microbiol. 2023 Feb 17;13:1127011. doi: 10.3389/fcimb.2023.1127011 (PMC9983756; doi:10.3389/fcimb.2023.1127011)
Supplement: Supplementary file 1 [file Presentation_1.pdf]

Supplemental Material

Disrupted Diurnal Oscillations of the Gut Microbiota in Patients with Alcohol Dependence

Kangqing Zhao, M.D.,[1, †] Zhaojun Ni, M.D.,[1, †] Ying Qin, M.D.,[2] Ran Zhu, M.D.,[1] Zhoulong Yu, Ph.D.,[1] Yundong Ma, M.D.,[1] Wenhao Chen, Ph.D.,[1] Qiqing Sun, M.Sc.,[1] Zhong Wang, Ph.D.,[1] Yanjing Liu, M.D.,[2] Jingwen Zhao, M.D.,[2] Wenjuan Peng, M.D.,[2] Sifan Hu, M.D.,[1] Jie Shi, M.D., Ph.D.,[3] Lin Lu, M.D., Ph.D.,[1] and Hongqiang Sun, M.D., Ph.D.[1,*]

[1]Peking University Sixth Hospital, Peking University Institute of Mental Health, NHC Key Laboratory of Mental Health (Peking University), National Clinical Research Center for Mental Disorders (Peking University Sixth Hospital), Beijing, 100191, China.

[2]The Second People's Hospital of Guizhou Province, Guizhou, 550004, China.

[3]National Institute on Drug Dependence and Beijing Key Laboratory of Drug Dependence, Peking University, Beijing, China; The State Key Laboratory of Natural and Biomimetic Drugs, Peking University, Beijing, China; The Key Laboratory for Neuroscience of the Ministry of Education and Health, Peking University, Beijing, 100191, China

[†]These authors contributed equally to this work.

*** Correspondence:**
Hongqiang Sun
sunhq@bjmu.edu.cn

1 Gut microbiota analysis

1.1 DNA extraction

The microbial community DNA was extracted using a MagPure Stool DNA KF Kit B (Magen, China) following the manufacturer's instructions. DNA was quantified with a Qubit Fluorometer by using a Qubit® dsDNA BR Assay Kit (Invitrogen, USA), and the quality was checked by electrophoresing aliquots on a 1% agarose gel.

1.2 Library construction

The variable region V4 of the bacterial 16S rRNA gene was amplified with the degenerate PCR primers 515F (5'-GTGCCAGCMGCCGCGGTAA-3') and 806R (5'-GGACTACHVGGGTWTCTAAT-3'). Both the forward and reverse primers were tagged with Illumina adaptor, pad and linker sequences. PCR enrichment was performed in a 50 μL reaction containing 30 ng of template, fusion PCR primer and PCR master mix. PCR

cycling conditions were as follows: 95 ℃ for 3 minutes; 30 cycles of 95 ℃ for 45 seconds, 56 ℃ for 45 seconds, and 72 ℃ for 45 seconds; and final extension for 10 minutes at 72 ℃. The PCR products were purified using Agencourt AMPure XP beads and eluted in elution buffer. Libraries were qualified by an Agilent Technologies 2100 bioanalyzer. The validated libraries were used for sequencing on the Illumina HiSeq 2500 platform (BGI, Shenzhen, China) following the standard pipelines of Illumina, which generated 2 × 250 bp paired-end reads.

1.3 Sequencing and bioinformatics analysis

Raw reads were filtered to remove adaptors and low-quality and ambiguous bases, and then paired-end reads were added to tags by the Fast Length Adjustment of Short reads program (FLASH, version 1.2.11) to obtain the tags (Magoc and Salzberg, 2011). The tags were clustered into OTUs with a cut-off value of 97% using UPARSE software (version 7.0.1090) (Edgar, 2013), and chimera sequences were compared against the Gold database using UCHIME (version 4.2.40) (Edgar et al., 2011) for detection. Then, OTU representative sequences were taxonomically classified using Ribosomal Database Project (RDP) Classifier version 2.2 with a minimum confidence threshold of 0.6 and were trained on the Greengenes database version 201305 by QIIME version 1.8.0 (Caporaso et al., 2010). USEARCH_global (Edgar, 2010) was used to compare all tags back to OTUs to obtain the OTU abundance statistics table of each sample. The α- and β-diversity were estimated by MOTHUR (version 1.31.2) (Schloss et al., 2009) and QIIME (version 1.8.0) (Caporaso et al., 2010), respectively, at the OTU level. α-Diversity was assessed using Chao1. β-Diversity was calculated using weighted UniFrac distance. Sample clustering was conducted by QIIME (version 1.8.0) (Caporaso et al., 2010) based on UPGMA. KEGG functions were predicted using PICRUSt software (Wilkinson et al., 2018).

Reference:

Caporaso, J.G., Kuczynski, J., Stombaugh, J., Bittinger, K., Bushman, F.D., Costello, E.K., et al. (2010). QIIME allows analysis of high-throughput community sequencing data. Nat. Methods 7, 335-336. doi: 10.1038/nmeth.f.303

Edgar, R.C. (2010). Search and clustering orders of magnitude faster than BLAST. Bioinformatics 26, 2460-2461. doi: 10.1093/bioinformatics/btq461

Edgar, R.C. (2013). UPARSE: highly accurate OTU sequences from microbial amplicon reads. Nat Methods 10, 996-998. doi: 10.1038/nmeth.2604

Edgar, R.C., Haas, B.J., Clemente, J.C., Quince, C. & Knight, R. (2011). UCHIME improves sensitivity and speed of chimera detection. Bioinformatics 27, 2194-2200. doi: 10.1093/bioinformatics/btr381

Magoc, T. & Salzberg, S.L. (2011). FLASH: fast length adjustment of short reads to improve genome assemblies. Bioinformatics 27, 2957-2963. doi: 10.1093/bioinformatics/btr507

Schloss, P.D., Westcott, S.L., Ryabin, T., Hall, J.R., Hartmann, M., Hollister, E.B., et al. (2009). Introducing mothur: open-source, platform-independent, community-

supported software for describing and comparing microbial communities. Appl. Environ. Microbiol. 75, 7537-7541. doi: 10.1128/aem.01541-09

Wilkinson, T.J., Huws, S.A., Edwards, J.E., Kingston-Smith, A.H., Siu-Ting, K., Hughes, M., et al. (2018). CowPI: A Rumen Microbiome Focussed Version of the PICRUSt Functional Inference Software. Front. Microbiol. 9, 1095. doi: 10.3389/fmicb.2018.01095

Table S1. Medications Prescribed in AD Patients During Fecal Sampling.

Patients	Medications	Dose (mg/day)	Duration of Medications	Patients	Medications	Dose (mg/day)	Duration of Medications
AD1	Lorazepam	6	4	AD19	Lorazepam	3	7
	Olanzepine	2.5	4		Quetiapine	100	7
	Vitamin B6	200	4		Valproate	1000	7
AD2	Bicyclol	150	4	AD20	Quetiapine	100	2
	Diazepam	20	4		Diazepam	20	2
	Olanzepine	5	4		Valproate	1000	2
	Vitamin B6	200	4	AD22	Lorazepam	6	2
AD3	Quetiapine	50	4		Quetiapine	50	2
	Oxazepam	45	4		Valproate	500	2
AD4	Diazepam	12.5	6	AD23	Fluvoxamine	150	2
	Lorazepam	4	6		Oxazepam	90	2
	Olanzepine	2.5	7		Valproate	500	2
	Vitamin B6	200	7	AD24	Lorazepam	3	2
AD6	Diazepam	20	3		Quatiapine	50	2
	Olanzepine	5	3	AD25	Diazepam	10	2
	Vitamin B6	200	3		Citalopram	10	2
AD7	Valproate	500	3		Quetiapine	150	2
	Olanzepine	5	3		Valproate	500	2
	Oxazepam	30	3	AD26	Oxazepam	30	2
AD8	Quetiapine	100	3		Quatiapine	50	2
	Lorazepam	3	3	AD28	Bicyclol	150	3
AD9	Risperidone	1	3		Diazepam	20	3
	Lorazepam	6	3		Olanzepine	5	3
AD11	Olanzepine	2.5	6		Valproate	500	3
	Diazepam	20	2	AD29	Aripiprazole	2.5	2
AD12	Bicyclol	150	3		Lorazepam	6	2
	Diazepam	20	3	AD30	Bicyclol	150	3
	Olanzepine	5	3		Lorazepam	6	3
	Valproate	500	3		Quetiapine	100	3
AD13	Diazepam	20	2		Valproate	1000	3
	Olanzepine	10	2	AD31	Diazepam	30	2
	Valproate	500	2		Risperidone	1	2
AD14	Lorazepam	3	4	AD33	Diazepam	20	3
	Olanzepine	5	4		Olanzepine	5	3
	Valproate	500	4		Valproate	1000	3
AD15	Diazepam	20	3	AD34	Bicyclol	150	2
	Olanzepine	5	3		Diazepam	30	2
	Valproate	500	3		Olanzepine	2.5	2
AD16	Olanzepine	5	3		Valproate	250	2
	Valproate	500	3	AD35	Diazepam	2	2
	Lorazepam	3	3		Olanzepine	5	2
AD17	Lorazepam	6	3	AD36	Diazepam	10	1
	Olanzepine	5	3		Olanzepine	5	1
AD18	Lorazepam	6	3	AD37	Diazepam	30	1
	Olanzepine	5	3		Olanzepine	5	1

Patients AD5, 10, 21, 27, 32 were excluded from the analysis due to the reasons depicted in Supplemental Figure S1.

Table S2. Fecal samplings of all the enrolled subjects

Year: 2019	Fecal Sampling Time Points			
**Date**	**7:00**	**11:00**	**15:00**	**19:00**
7-18				HC01
7-19	HC01			
7-20	AD01		HC01	
7-21		AD01, HC01		
7-22	AD02	AD03	AD01	AD02
7-23	AD03, AD04	AD02, AD04	AD02, AD04	AD01, AD03
7-24	AD06, AD07		AD03	AD04, AD06, AD07
7-25		AD07	AD06, AD07	
7-26		AD06, AD08		AD08
7-27	AD08	AD09, HC02	AD08	AD09, HC02
7-28	AD09, HC02		AD09, HC02	
7-29				
7-30	AD11, HC03		AD11, HC03	AD12, HC04
7-31	AD12	AD11, HC03, HC04	AD12, HC04	AD11
8-1	HC04	AD12		HC03
8-2				HC05, HC06
8-3	HC06	HC05	HC06	
8-4		HC06	HC05	
8-5				
8-6	HC05	HC08		HC08, HC09
8-7	HC08	HC09	HC08	
8-8	HC09	AD13	HC09	AD13
8-9			AD13	HC12
8-10	AD13, HC11, HC12		HC11, HC12	HC10
8-11	AD15, HC10, HC13	AD14, AD16, HC11, HC12	AD15, HC10, HC13	AD16, HC11
8-12	AD16, HC14	AD15, HC10, HC13	AD14, AD16, HC14	AD14, AD15
8-13	AD14, HC15	HC14	HC15	HC13, HC14, HC15
8-14		AD17, HC15		HC16
8-15	AD17, HC16		AD17, AD18, HC16	AD17, AD18, HC17
8-16	AD18, AD19, HC17	AD18, HC16, HC17	HC17	
8-17		AD19	AD19	AD19
8-18				
8-19				HC19, HC20
8-20	AD20, HC18, HC19, HC20	AD20, HC19	AD20, HC18, HC19, HC20	AD20, HC18
8-21		HC18, HC20		HC21
8-22	HC21			
8-23		HC21	HC21	
8-24	The staffs engaged in the fecal samples collection in our study were taking the national qualification examination for medical practitioners, and therefore, no subjects were enrolled during this time.			
8-25				
8-26				
8-27				
8-28			AD22	AD22
8-29		AD22		AD23
8-30	AD22		AD23	
8-31		AD23		
9-1	AD21, AD23		AD24	AD24
9-2	AD24	AD24, AD25, AD26	AD21, AD26	AD25
9-3	AD26	AD21	AD25	AD26
9-4	AD25		AD28, AD30	AD28
9-5	AD28, AD29	AD28, AD29		
9-6		AD30	AD29	AD29, AD30, AD31
9-7	AD31	AD31	AD31, AD33	
9-8	AD30			AD33
9-9	AD33	AD33		
9-10	No qualified subjects were enrolled during this time.			
9-11				
9-12				

9-13				
9-14				
9-15				
9-16		AD34	AD34	AD34
9-17				
9-18	AD34			
9-19	No qualified subjects were enrolled during this time.			
9-20				
9-21				
9-22				
9-23				
9-24				AD35
9-25	AD35		AD35	
9-26		AD35		
9-27				
9-28	AD36	AD36, AD37	AD36, AD37	AD36, AD37
9-29	AD37			
Additional information				

1. The left column is the date starting from July 18 to September 29, and the top row indicates the four sampling time points, so each cell represents the time point on the day. The cells are filled with subjects that defecated at the corresponding time point on the corresponding day.
2. The empty cells in this table mean that there are not available fecal samples collected at the corresponding time point.
3. The subject AD05 was excluded due to acute pancreatitis; the subject AD10 was excluded due to constipation, and unable to defecate on time; the subject AD21 was unable to provide the fecal sample at 19:00, and therefore, was excluded; the subject AD27 was suddenly exhibiting psychotic symptoms, and was excluded; the subject AD32 was having diarrhea and was excluded.

AD, alcohol dependence; HC, healthy control

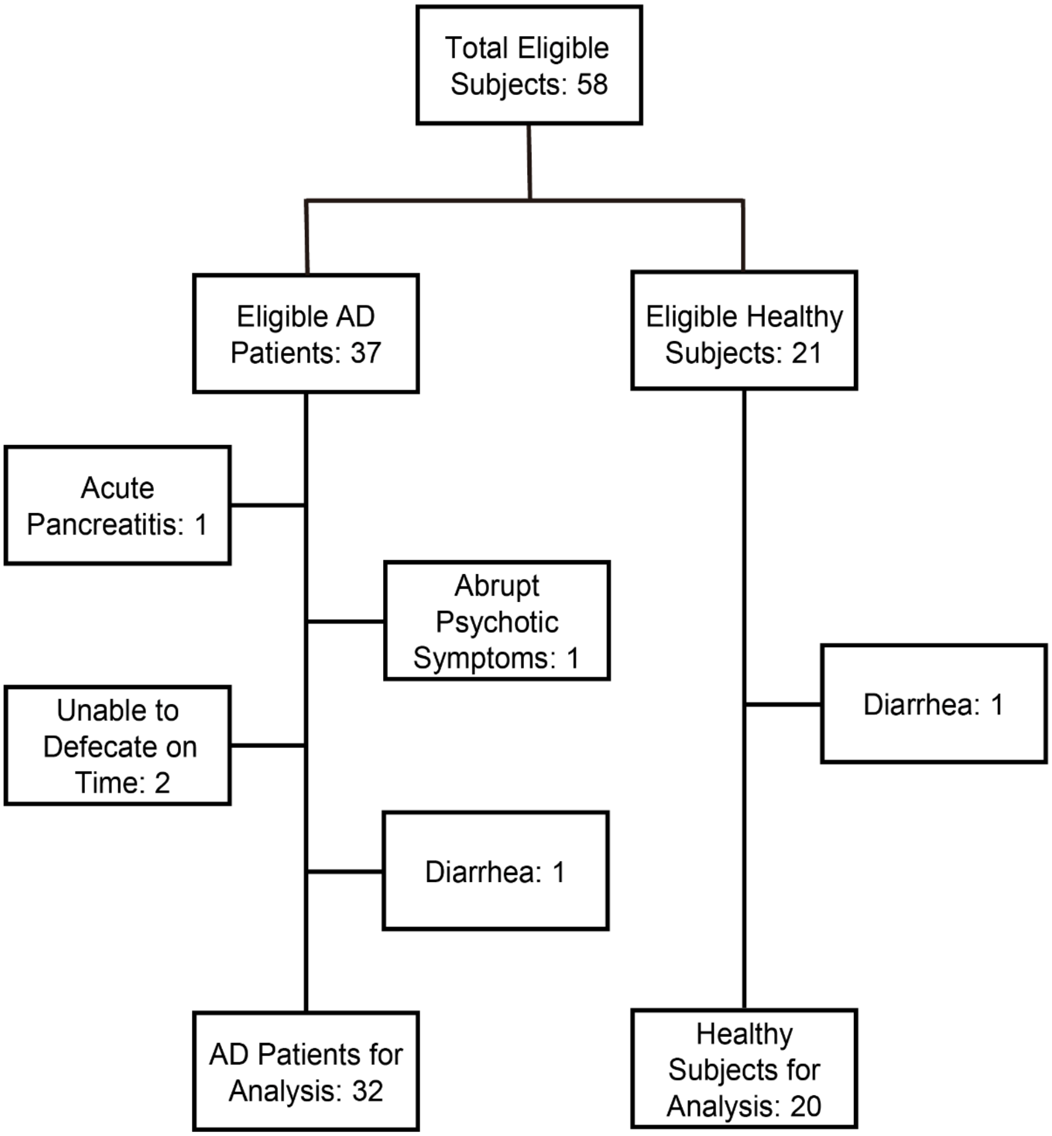

Figure S1. Flowchart of Participants in the Study.

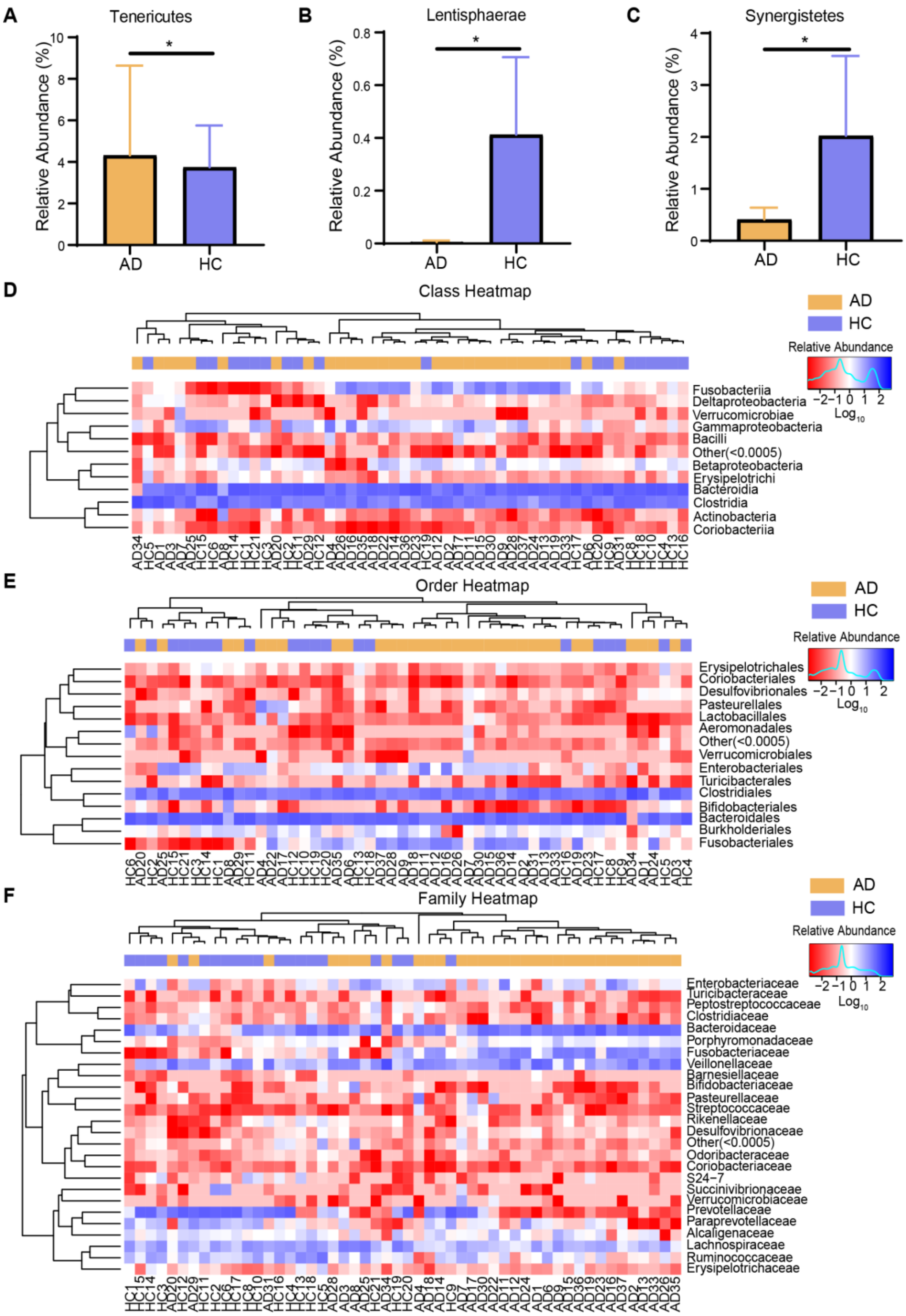
A
Tenericutes
Relative Abundance (%)
AD
HC
B
Lentisphaerae
C
Synergistetes
D
Class Heatmap
Relative Abundance
Log10
Fusobacteriia
Deltaproteobacteria
Verrucomicrobiae
Gammaproteobacteria
Bacilli
Other(<0.0005)
Betaproteobacteria
Erysipelotrichi
Bacteroidia
Clostridia
Actinobacteria
Coriobacteriia
E
Order Heatmap
Erysipelotrichales
Coriobacteriales
Desulfovibrionales
Pasteurellales
Lactobacillales
Aeromonadales
Other(<0.0005)
Verrucomicrobiales
Enterobacteriales
Turicibacterales
Clostridiales
Bifidobacteriales
Bacteroidales
Burkholderiales
Fusobacteriales
F
Family Heatmap
Enterobacteriaceae
Turicibacteraceae
Peptostreptococcaceae
Clostridiaceae
Bacteroidaceae
Porphyromonadaceae
Fusobacteriaceae
Veillonellaceae
Barnesiellaceae
Bifidobacteriaceae
Pasteurellaceae
Streptococcaceae
Rikenellaceae
Desulfovibrionaceae
Other(<0.0005)
Odoribacteraceae
Coriobacteriaceae
S24-7
Succinivibrionaceae
Verrucomicrobiaceae
Prevotellaceae
Paraprevotellaceae
Alcaligenaceae
Lachnospiraceae
Ruminococcaceae
Erysipelotrichaceae

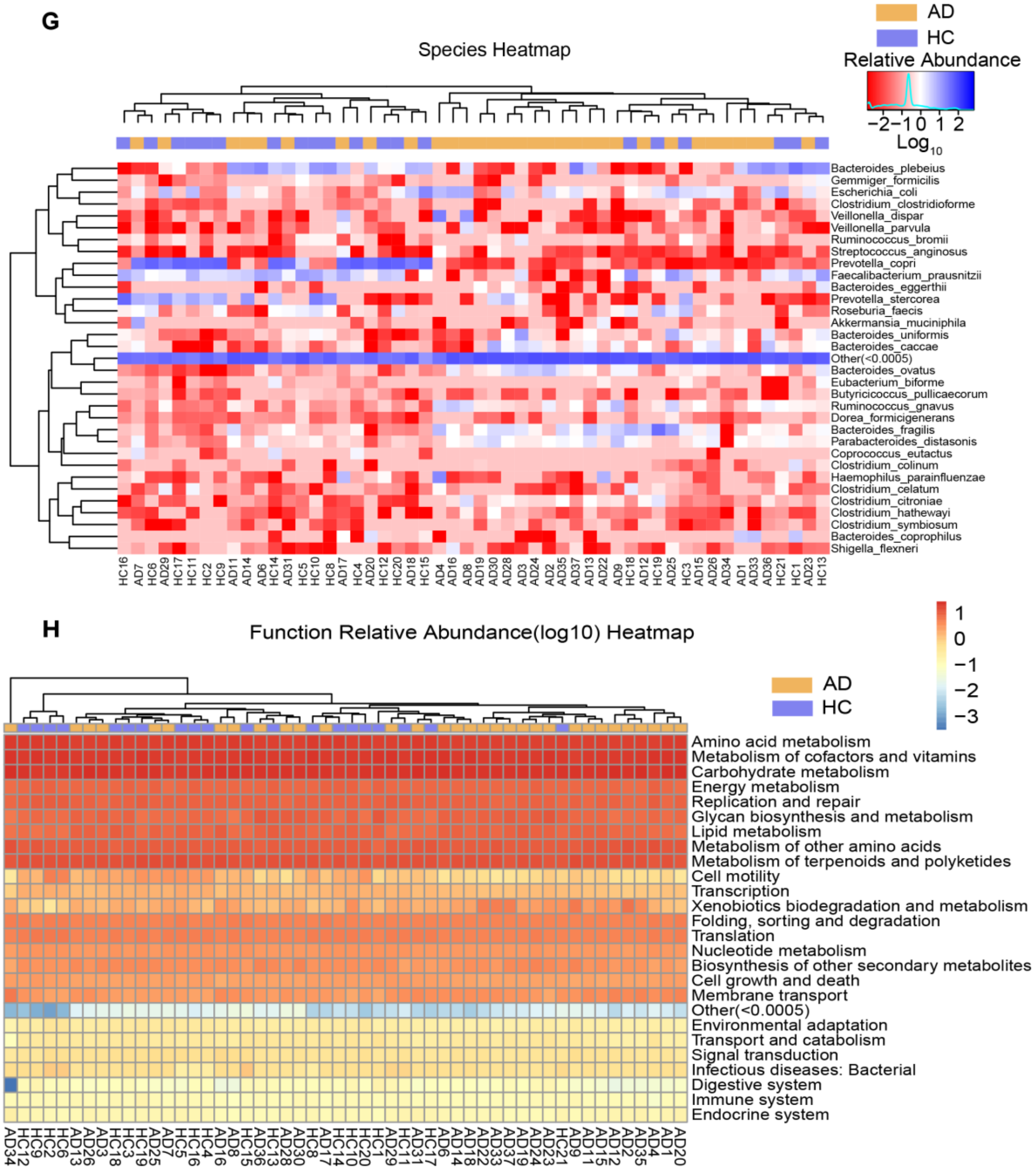

Figure S2. Alterations of the Gut Microbiota in AD Patients, Related to Figure 1. (**A-C**) The relative abundance of Tenericutes, Lentisphaerae and Synergistetes in AD patients and healthy subjects. (**D-G**) Heatmap representation of the most prevalent classes, orders, families and species in the gut microbiota of AD patients and healthy subjects (the columns correspond to the subjects; the groups are denoted with a top color bar; the figure shows the taxa with a relative abundance of ≥ 0.05%; the hierarchical clustering was performed using the Euclidean metric and complete linkage). (**H**) Heatmap representation of the most prevalent KEGG pathways in the gut microbiota of AD patients and healthy subjects. Data are expressed as the mean ± SEM. *$p < 0.05$.

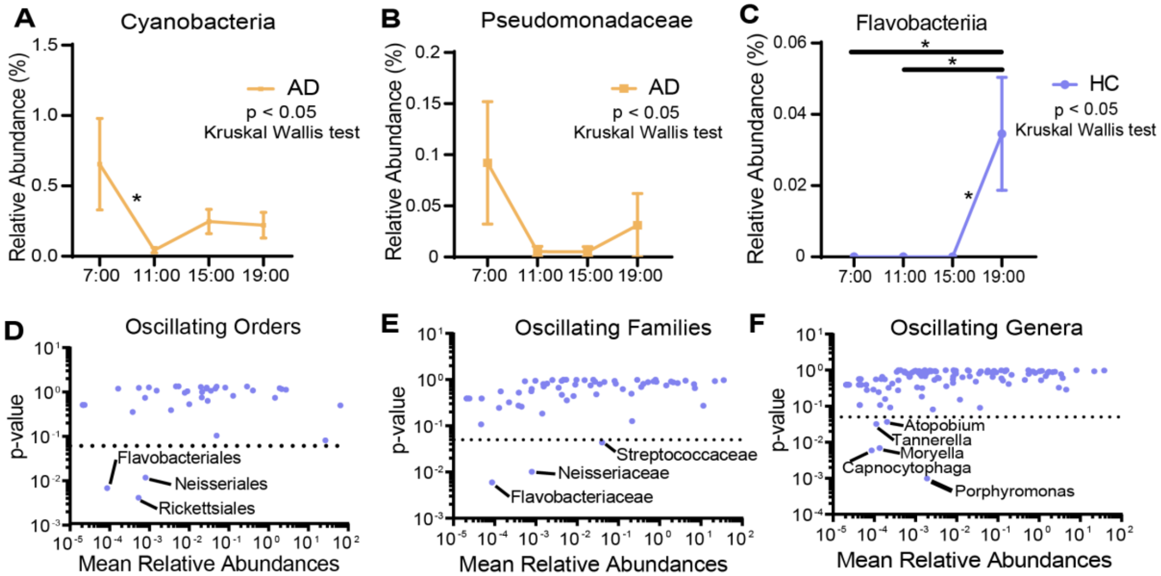
A
Cyanobacteria
Relative Abundance (%)
AD
p < 0.05
Kruskal Wallis test
7:00 11:00 15:00 19:00
B
Pseudomonadaceae
Relative Abundance (%)
AD
p < 0.05
Kruskal Wallis test
7:00 11:00 15:00 19:00
C
Flavobacteriia
Relative Abundance (%)
HC
p < 0.05
Kruskal Wallis test
7:00 11:00 15:00 19:00
D
Oscillating Orders
p-value
Flavobacteriales
Neisseriales
Rickettsiales
Mean Relative Abundances
E
Oscillating Families
p-value
Streptococcaceae
Neisseriaceae
Flavobacteriaceae
Mean Relative Abundances
F
Oscillating Genera
p-value
Atopobium
Tannerella
Moryella
Capnocytophaga
Porphyromonas
Mean Relative Abundances

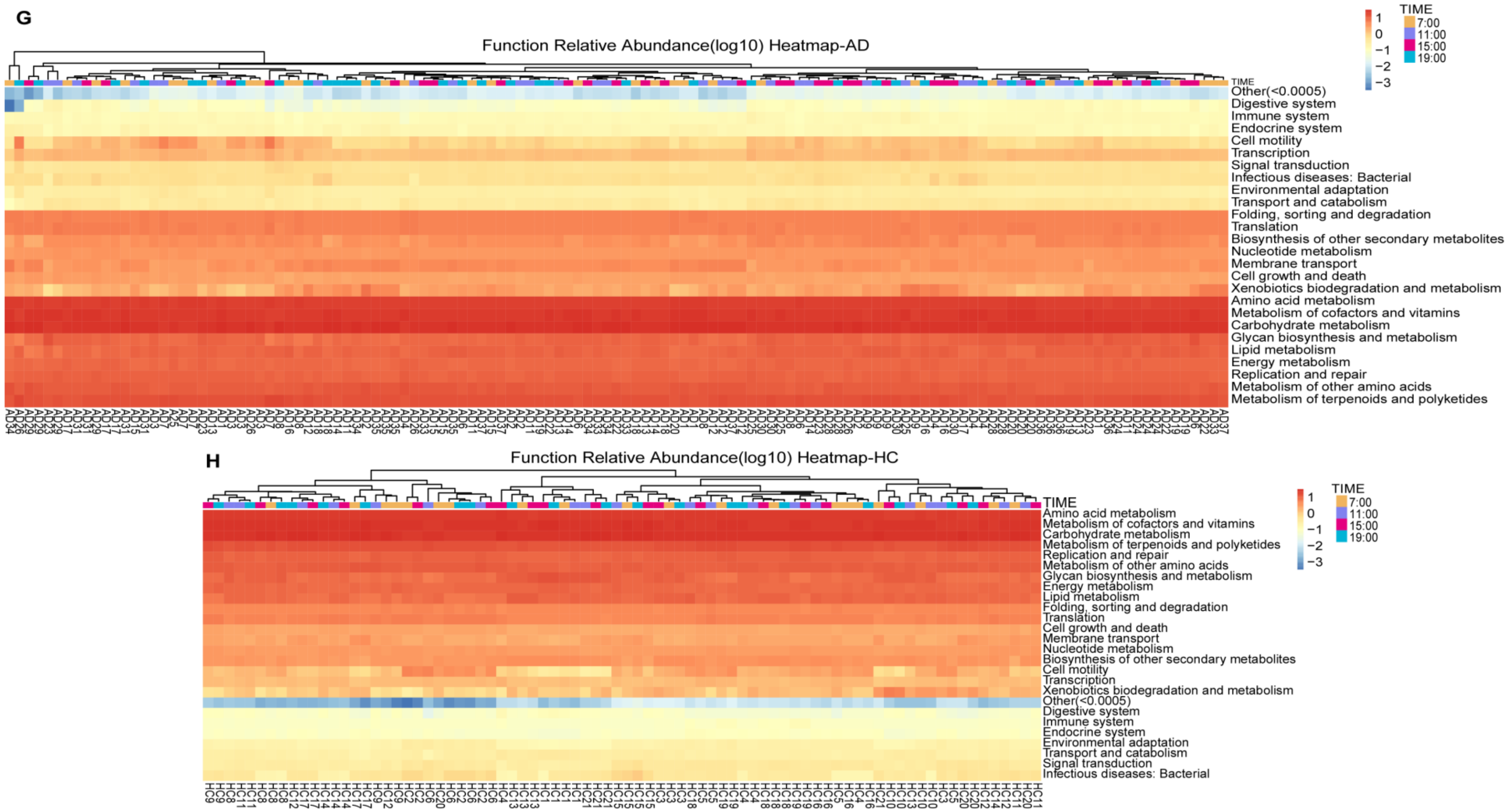
G
Function Relative Abundance(log10) Heatmap-AD
TIME
7:00
11:00
15:00
19:00
1
0
−1
−2
−3
Other(<0.0005)
Digestive system
Immune system
Endocrine system
Cell motility
Transcription
Signal transduction
Infectious diseases: Bacterial
Environmental adaptation
Transport and catabolism
Folding, sorting and degradation
Translation
Biosynthesis of other secondary metabolites
Nucleotide metabolism
Membrane transport
Cell growth and death
Xenobiotics biodegradation and metabolism
Amino acid metabolism
Metabolism of cofactors and vitamins
Carbohydrate metabolism
Glycan biosynthesis and metabolism
Lipid metabolism
Energy metabolism
Replication and repair
Metabolism of other amino acids
Metabolism of terpenoids and polyketides
H
Function Relative Abundance(log10) Heatmap-HC
TIME
7:00
11:00
15:00
19:00
1
0
−1
−2
−3
Amino acid metabolism
Metabolism of cofactors and vitamins
Carbohydrate metabolism
Metabolism of terpenoids and polyketides
Replication and repair
Metabolism of other amino acids
Glycan biosynthesis and metabolism
Energy metabolism
Lipid metabolism
Folding, sorting and degradation
Translation
Cell growth and death
Membrane transport
Nucleotide metabolism
Biosynthesis of other secondary metabolites
Cell motility
Transcription
Xenobiotics biodegradation and metabolism
Other(<0.0005)
Digestive system
Immune system
Endocrine system
Environmental adaptation
Transport and catabolism
Signal transduction
Infectious diseases: Bacterial

Figure S3. Diurnal Oscillation of the Gut Microbiota, Related to Figure 2. (**A-B**) Relative abundance of Cyanobacteria and Pseudomonadaceae at 4 different time points in AD patients. (**C**) Relative abundance of Flavobacteriia at 4 different time points in healthy subjects. (**D-F**) Orders, families and genera showing diurnal oscillation in healthy subjects. (**G-H**) Heatmap representation of the most prevalent KEGG pathways oscillating among 4 different time points in AD patients and healthy subjects. Data are expressed as the mean ± SEM. *$p < 0.05$.

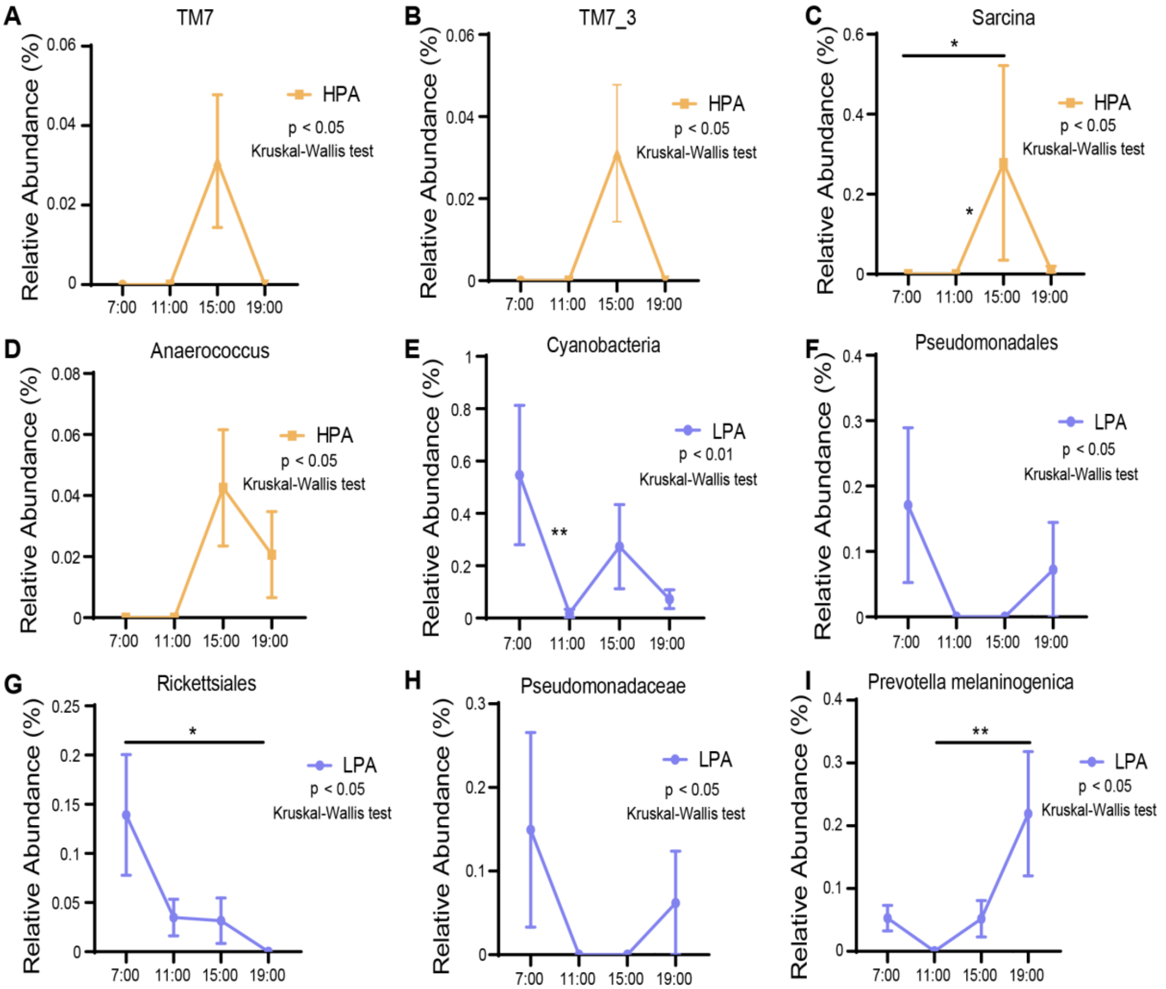

Figure S4. Diurnal Oscillation of the Gut Microbiota, Related to Figure 6. (**A-D**) Relative abundances of TM7, TM7_3, *Sarcina* and *Anaerococcus* at 4 different time points in the HPA group. (**E-I**) Relative abundances of Cyanobacteria, Pseudomonadales, Rickettsiales, Pseudomonadaceae and *Prevotella melaninogenica* at 4 different time-points in the LPA group. Data are expressed as the mean ± SEM. *$p < 0.05$.
